# Retraction: Beltaïef *et al.* An Expeditious Synthesis of [1,2]Isoxazolidin-5-ones and [1,2]Oxazin-6-ones from Functional Allyl Bromide Derivatives. *Molecules* 2010, *15*, 4094-4101

**DOI:** 10.3390/molecules191016810

**Published:** 2014-10-17

**Authors:** Martyn Rittman

**Affiliations:** MDPI AG, Klybeckstrasse 64, Basel CH-4057, Switzerland; E-Mail: rittman@mdpi.com

We have been made aware that the figures, tables, compounds and experimental data reported in the title paper [1] are duplicated in another publication by the same authors [2]. Comparing the received dates of both articles, it is apparent that, although the *Molecules* article was published more than a year earlier, it was submitted to MDPI several weeks after the manuscript of [2] was received by Taylor and Francis. Since it is our policy to only consider for publication original unpublished articles that are not under consideration by another journal, and do not potentially infringe any copyright, and all authors explicitly agree to this as a condition for processing their submissions, we view this as a deliberate attempt to double publish this material and a violation of our rules on this matter. MDPI is a member of the Committee on Publication Ethics and takes the responsibility to enforce strict ethical policies and standards very seriously. To ensure the addition of only high quality scientific works to the field of scholarly publication, paper [1] is retracted and shall be marked accordingly. We apologize to our readership that this went undetected until now.
